# Therapeutic effect of local photothermal heating of gold nanoparticle-coated self-expandable metallic stents for suppressing granulation tissue formation in the mouse colon

**DOI:** 10.1371/journal.pone.0249530

**Published:** 2021-04-02

**Authors:** Yeong-Cheol Heo, Dong-Kyoon Han, Min Tae Kim

**Affiliations:** 1 Department of Radiological Science, College of Health Science, Eulji University, Seongnam, South Korea; 2 Department of Radiologic Technology, Cheju Halla University, Jeju-si, Jeju-do, South Korea; 3 Biomedical Engineering Research Center, Asan Medical Center, University of Ulsan College of Medicine, Songpa-gu, Seoul, South Korea; Massachusetts General Hospital, UNITED STATES

## Abstract

**Purpose:**

To investigate the therapeutic effect of local photothermal (PT) heating on suppression of stent-induced granulation tissue formation in mouse colon.

**Materials and methods:**

A gold nanoparticle (GNP)-coated self-expandable metallic stent (SEMS) was prepared using a two-step synthesis process for local PT heating under near-infrared laser irradiation. Twenty-four mice were randomly divided into two groups of 12 and subjected to SEMS placement in the colon. Group A received a GNP-coated SEMS without local heating and Group B received a GNP-coated SEMS and underwent local heating at 55°C after SEMS placement. The therapeutic effect of local heating was assessed by comparing the histopathological, immunohistochemical, and endoscopic results.

**Results:**

Four mice were excluded because of stent migration (n = 3, group B) or death (n = 1, group A). Stent-induced granulation tissue-related variables were significantly lower in group B than in group A (p < 0.001). *In vivo* endoscopic images, 4 weeks after stent placement, showed granulation tissue formation over the wire mesh in group A and relatively good patency of the stented colon with no definite irregularities in group B. There was more vascular endothelial growth factor (VEGF) positivity in group A than in group B.

**Conclusion:**

Local PT heating suppresses granulation tissue formation after stent placement in mouse colon.

## Introduction

Self-expandable metallic stent (SEMS) placement was originally developed for treating malignant and benign strictures [1–7]. Colonic stent placement is effective and safe and can be used as a palliative treatment or an alternative to surgery [6–10]. Stents are commonly used to treat diverticular disease, Crohn’s disease, colonic fistula, and postsurgical anastomosis [11–19]. However, therapeutic options for these patients are restricted by the stent-induced granulation tissue formation that occurs in the uncovered portion of the SEMS or the ends of covered SEMS; it can induce additional stricture with recurrent obstruction and increase the technical failure of stent removal [7]. Therefore, uncovered and covered SEMS placement is insufficient for patients with benign and malignant strictures, and stenting for benign strictures is not yet recommended as a first treatment option. Various drugs such as paclitaxel, sirolimus, and transforming growth factor-beta inhibitors have been investigated *in vitro* and *in vivo* for their ability to suppress granulation tissue formation after SEMS placement in various nonvascular, luminal organs [20–23]. However, despite recent advances in various stent technologies for suppressing stent-induced granulation tissue formation, the current therapeutic strategies are insufficient. Additionally, there is a lack of data on long-term outcomes.

Laser-induced local heating can suppress tumor and granulation tissue formation [24–28]. Under near-infrared (NIR) laser-induced local heating, the temperature of the gold nanoparticle (GNP)-coated stent is significantly increased leading to hyperthermia of the tissue [27–30]. Local heating at moderate temperatures reduces collagen deposition, increases apoptosis, and activates heat shock proteins [25–27]. Various GNP-based stents with multifunctionality and high photothermal therapy efficiency have been investigated [28,31–33]. Our previous study using GNP-coated SEMS with near-infrared (NIR) irradiation demonstrated that *in vivo* temperatures (49°C) can suppress the stent-induced tissue granulation tissue formation [27]. However, thermal effects did not improve the suppression of hyperplasia or endoscopic tissue changes. A moderate temperature may suppress the revascularization of stented mechanical injury. We hypothesized that this therapeutic strategy may also suppress stent-induced granulation tissue formation by thermal-induced suppression of revascularization. The aim of this study was to investigate the therapeutic effect of local photothermal (PT) heating on suppressing stent-induced granulation tissue formation in the mouse colon.

## Materials and methods

### Preparation of GNP-coated SEMS

**Fig 1:**
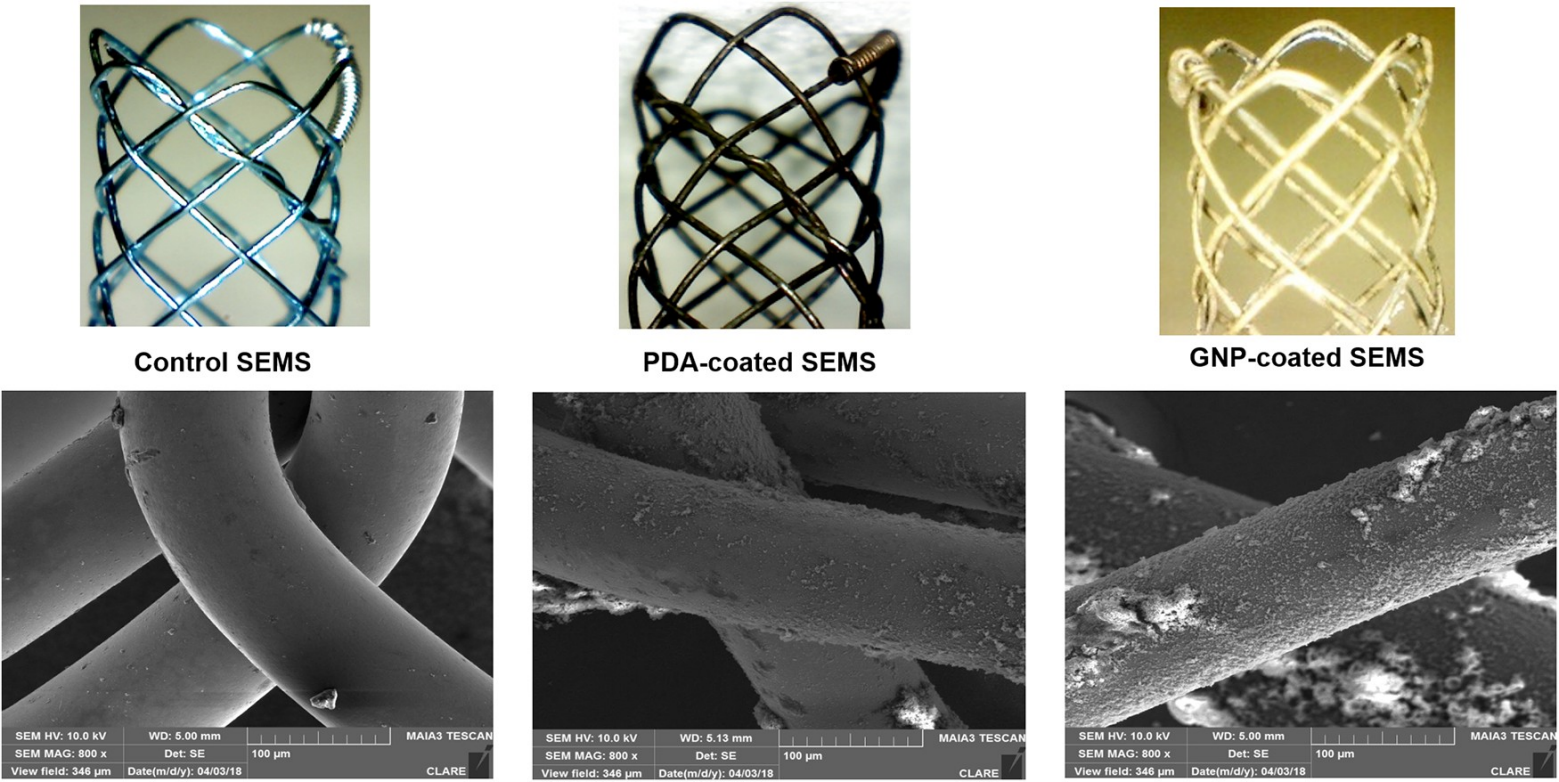
Representative scanning electron microscopy images after the stent coating process. PDA: Polydopamine; GNP: Gold nanoparticle.

The SEMS was knitted from a single 0.127-mm-thick nitinol wire filament (S&G Biotech, Yongin, South Korea). The stent was 4 mm in diameter and 8 mm in length. The colonic stent introducer set consisted of a 6-Fr sheath, dilator, and pusher catheter (Cook, Bloomington, IN, USA). The materials for the preparation of gold-coated stents have been previously described [27,34]. The preparation of the GNP-coated stent and its characterization were performed as previously described [27,34] (Fig 1). GNP-coated SEMS was fabricated through a two-step synthesis process to create a stent capable of PT local heating under NIR laser irradiation. The cationic polymer was coated on the surface of SEMS through polydopamine (PDA) coating before deposition of GNP on the surface. To coat the surface of the PDA-coated stent with a cationic polymer, the PDA-coated stent was immersed in PEI. Finally, the PEI-coated stent was immersed to synthesize the gold nanoparticle.

### Animal study

All animal experiments were approved by the Institutional Animal Care and Use Committee at the Asan Medical Center, University of Ulsan College of Medicine (No. 2017-13-267). The animals were housed one per cage in a room with 12-hour light/dark cycles at an environmental temperature (24 ± 1°C) and moisture (55 ± 10%); they were provided standard rodent chow and water ad libitum. All animals were acclimatized for at least 1 week before the experiment. Twenty-four C57BL/6 male mice (25–30 g; Orient Bio, Seongnam, South Korea) were divided using a random allocation software (version 2.0; Microsoft, Seattle, WA, USA) into two groups: Group A, GNP-SEMS without local heating, and Group B, GNP-SEMS with local heating at 55°C. All mice were euthanized by inhalable pure carbon dioxide 4 weeks after the start of local heating, which was started 1 week after SEMS placement.

### Stent placement and local PT heating

**Fig 2:**
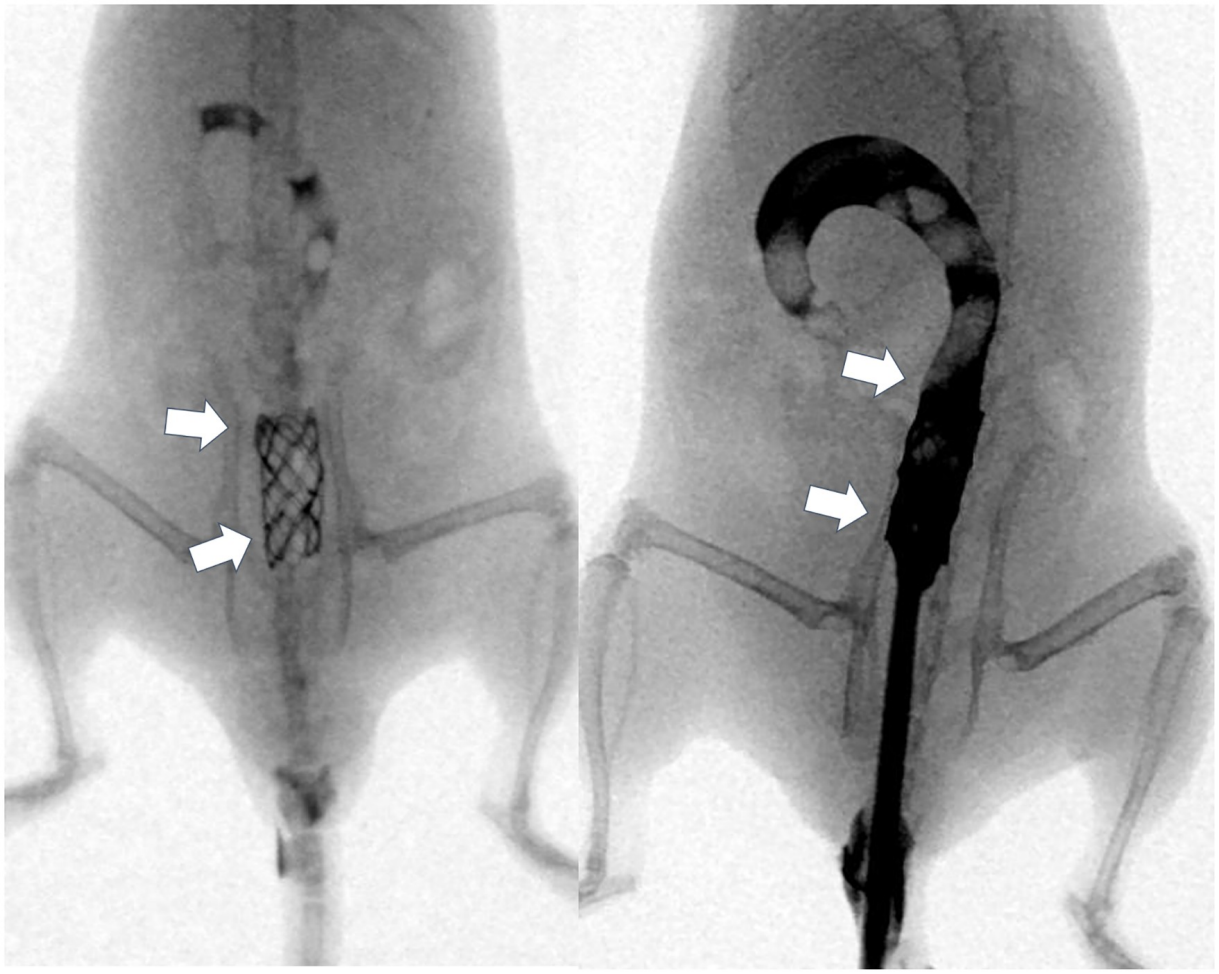
Representative radiographic images showing the stent in the sigmoid colon at the level of the pelvis. [Arrows = stented sigmoid colon at the level of the pelvis].

All procedures were performed on a heating mat warmed to 38°C with the mouse in the supine position. The mice were anesthetized by intramuscular injection of 50 mg/kg zolazepam, 50 mg/kg tiletamine (Zoletil 50; Virbac, Carros, France), and 10 mg/kg xylazine (Rompun; Bayer HealthCare, Leverkusen, Germany). A 0.014-inch micro-guidewire (Transcend; Boston Scientific, Watertown, MA, USA) was inserted under fluoroscopic guidance, and a 4-Fr sheath and dilator were advanced over the guidewire into the sigmoid colon at the level of the pelvis. With the sheath left in place, the guidewire and dilator were removed from the mouse. A GNP-coated SEMS in a compressed state was loaded into the sheath and placed in the sigmoid colon using a pusher catheter. The GNP-coated SEMS was deployed at the level of the upper pelvis under fluoroscopic monitoring. After the procedure, colonography was performed to verify the position and patency of the stent (Fig 2).

Local PT heating was performed 1 week after the stent placement. For NIR laser irradiation, a 1-mm-diameter fiber-coupled NIR (808 nm) diode laser (OCLA^™^ LASER, NDLUX Inc., Anyang, South Korea) was inserted into a 6-Fr sheath with a radiopaque tip to allow visualization under fluoroscopic guidance. The 6-Fr sheath with the fiber-coupled NIR laser was advanced to the middle portion of the stented sigmoid colon. NIR laser irradiation was applied for 70 s (including 10 s after irradiation) in group B. Mice received an intramuscular injection of 0.05 mg/kg buprenorphine (Renophan; Hanlim Pharmaceutical, Seoul, South Korea) before the stent placement, local heating, and on days 1 and 2 after the interventional procedure. After the stent placement, weight change and behavioral change were monitored weekly. Four weeks after stent placement, all mice were sacrificed for histologic analysis. All mice were euthanized 4 weeks by inhalable pure carbon dioxide after stent placement.

#### *In vivo* endoscopic examination after PT laser-induced local heating

We evaluated tissue effects, such as the recovery of the epithelium and granulation tissue formation, after the stent placement and local heating in all mice.

Endoscopic evaluation using a Hopkins II rigid endoscope (Karl Storz, Goleta, CA, USA) was used to identify the adjacent stent mesh framework. *In vivo* endoscopic images were obtained 4 weeks after stent placement in Group A and immediately after local heating and 4 weeks after the stent placement in Group B.

*Histologic evaluation*. All mice were euthanized by inhalable pure carbon dioxide 4 weeks after stent placement. Surgical exploration of the colon was followed by a gross examination to evaluate the degree of granulation tissue formation. The stents were then gently removed from the stented colon. The stented colons were sectioned transversely at the proximal and distal regions.

Tissue proximal region samples were fixed in 10% neutral-buffered formalin for 24 h and embedded in polymethyl methacrylate (Polysciences Inc, Warrington, PA, USA), which is a hard acrylic resin. Samples were then cut into sections using a tungsten carbide knife, leaving the stent wires intact in the cross-sections to minimize potential artifacts from stent wire removal.

Histological evaluation using hematoxylin and eosin included determining the thickness of submucosal fibrosis (mm) and the granulation tissue-related percentage of the stent. The cross-sectional area of stenosis was calculated as 100 × (1 - [stenotic stented area/original stented area]) [21,27]. Histological analysis of the colon was performed using a BX51 microscope (Olympus, Tokyo, Japan). Image-Pro Plus software (Media Cybernetics, Silver Spring, MD, USA) was used for the measurements. The analyses of the histologic findings were assessed based on the consensus of three observers blinded to the study.

### Immunohistochemical analysis

Formalin-fixed, paraffin-embedded sections of the colon were used for immunohistochemical analysis. Immunohistochemistry (IHC) was performed on paraffin-embedded transverse sections of the stented colon with VEGF (ab45010, 1:100, Abcam, Cambridge, UK) and as the primary antibodies. The sections were visualized using a BenchMark XT IHC automated immunohistochemical Stainer (Ventana Medical Systems, Tucson, AZ, USA). VEGF positivity were determined as follows: 1 = mild, 2 = mild to moderate, 3 = moderate, 4 = moderate to severe, and 5 = severe. IHC reporting was based on the consensus of three observers, who were blinded to the study.

### Statistical analysis

Differences between the groups were analyzed using the Mann-Whitney U test, as appropriate. A *p*-value of < 0.05 was considered statistically significant. Statistical analyses were performed using SPSS (version 24.0; SPSS, IBM, Chicago, IL, USA).

## Results

### Stent placement, local heating, and *in vivo* endoscopic findings

Stent placement and local PT heating were technically successful in all the mice (Fig 2). Four mice were excluded because of stent migration (n = 3) or death (n = 1). GNP-coated SEMSs had migrated into the rectums of three mice in group B within 10 days after placement. One mouse in group A died after stent placement because of perforation caused by the radial stent force in the colon. Data from these mice were omitted. The remaining 20 (83.3%) mice survived until the end of the experiment without stent-related complications.

**Fig 3:**
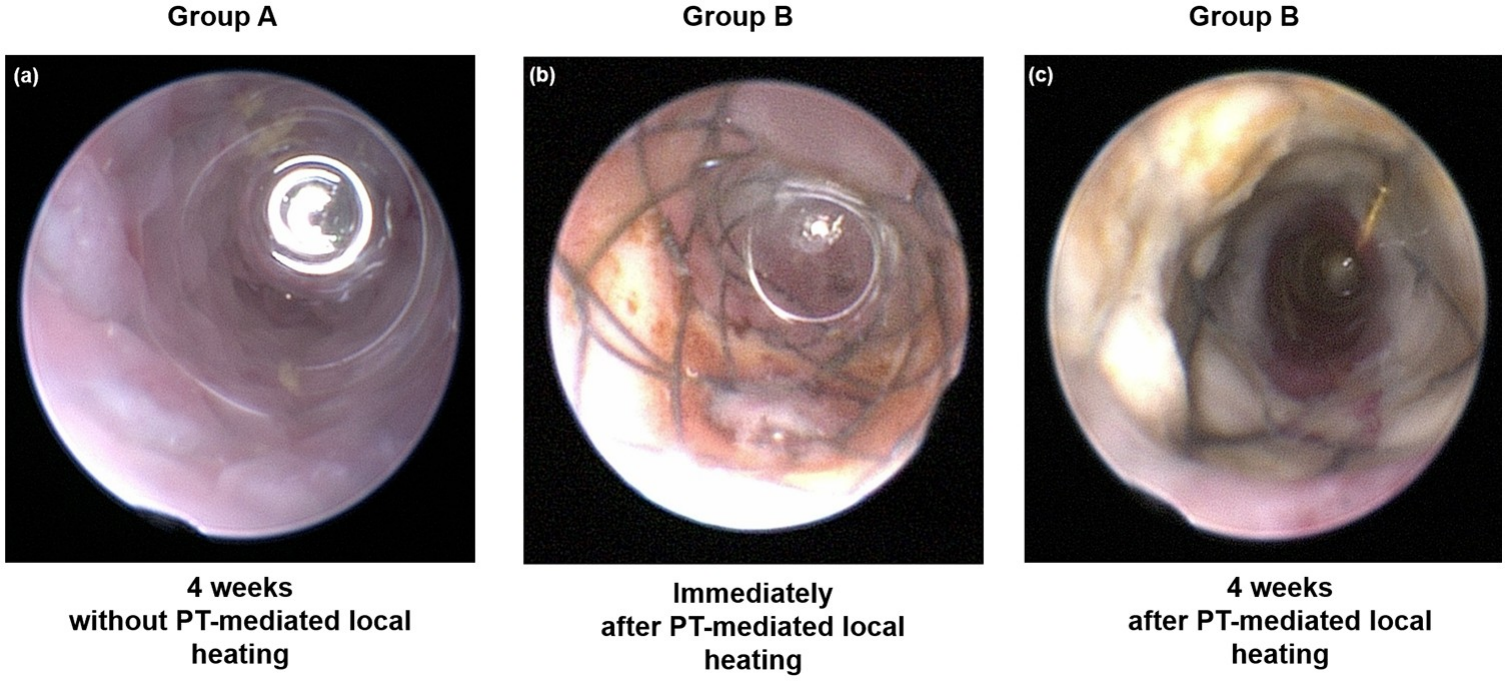
*In vivo* endoscopic images obtained (a) 4 weeks after stent placement without local heating in Group A, (b) immediately after local heating in Group B, and (c) 4 weeks after stent placement with local heating in Group B. PT: Photothermal.

The *in vivo* endoscopic images are shown in Fig 3a–3c. The colon mucosa adjacent to the stent wire was mildly burned in Group B immediately following local PT heating. Follow-up *in vivo* endoscopic images 4 weeks after stent placement showed granulation tissue formation over the wire mesh in Group A and relatively good patency of the stented colon, including the wire mesh, and no definite irregularities in Group B.

### Histologic findings

**Fig 4:**
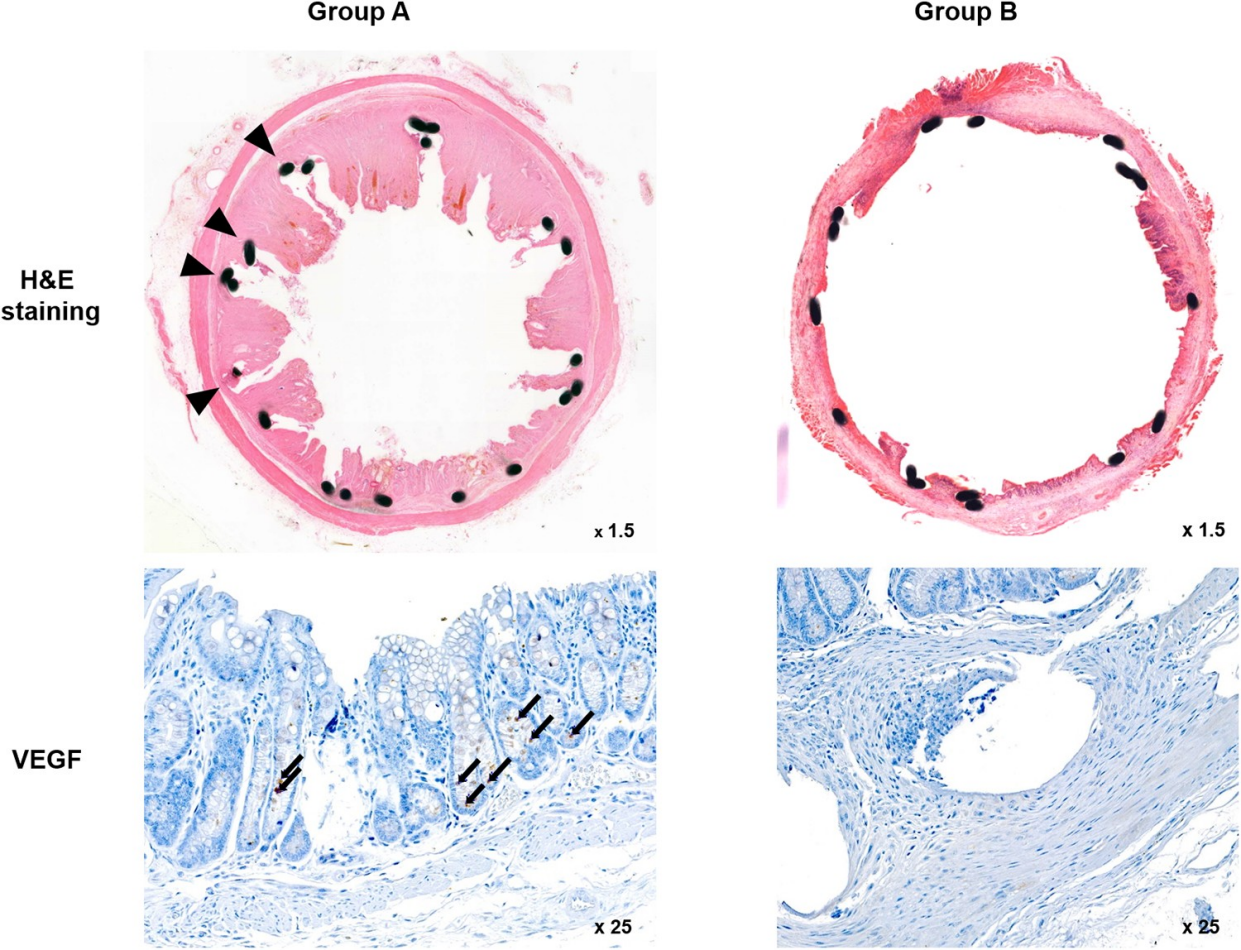
Representative microscopic images of histological sections and immunohistochemistry sections were obtained 4 weeks after stent placement. Hematoxylin and eosin (H&E) staining and VEGF immunohistochemistry. [Arrowheads = stent struts (magnification ×1.25), Arrows = VEGF-positive cells (magnification ×25)]. VEGF: Vascular endothelial growth factor.

The histological findings are summarized in Table 1 and examples are presented in Fig 4. The mean percentage of granulation tissue area, mean thickness of submucosal fibrosis, and mean VEGF positivity were significantly different between the groups (all *p* < 0.001, Mann-Whitney U test).

**Table 1:** Histological and immunohistochemical findings after GNP-coated stent placement with or without local heating.

	Group A	Group B	^+^*p*-value
**Granulation tissue area (%)**	49.80 ± 7.80	28.1 ± 7.07	0.001
**Thickness of submucosal fibrosis (mm)**	0.53 ± 0.17	0.26. ± 0.62	0.001
**VEGF positivity (%)**	3.72 ± 0.46	2.22 ± 0.44	0.001

Note. Data are presented as mean ± standard deviation.

^**+**^ Mann–Whitney U test.

GNP: Gold nanoparticle; VEGF: Vascular endothelial growth factor.

The mean percentage of the granulation tissue area and the mean thickness of submucosal fibrosis were significantly higher in Group A than in Group B. Further, immunohistochemical analysis showed that the degree of VEGF positivity was higher in Group A, which did not undergo local PT heating, than in Group B, which underwent local PT heating. Therefore, the level of VEGF decreased after local PT heating.

## Discussion

The minimally invasive intervention of colonic stricture using a stent strategy is currently limited due to the development of stent-induced granulation tissue formation. In this study, we investigated the therapeutic effect of local heating of GNP-coated SEMS, which generates significant heat when irradiated with a NIR laser. The stented sigmoid colon was successfully heated, as evidenced by the prominent burning change in the endoscopic evaluation. Granulation tissue formation-related histopathological features were decreased in mice treated with local PT heating but not in mice who received stenting alone. Further, hypervascularization was significantly decreased in Group B than in Group A. decrease in microvessels showed that stent-induced granulation tissue formation is associated with angiogenesis [35,36]. Our results demonstrate that local PT heating of the GNP-coated SEMS using NIR laser irradiation suppressed the stent-induced granulation tissue formation in the mouse colon.

The GNP-coated SEMS for local heating was synthesized using a two-step process. Covering the surface of the stent with GNPs is crucial for the *in vivo* heat effect. The GNP-coated SEMS was irradiated with a NIR laser; the burn change induced by the heat was then evaluated endoscopically to confirm the heat effect. Angiogenesis was also different between the two groups. Park et al. reported that PT can supply adequate heat to the stent, which helps suppress the stent-induced granulation tissue formation [27,30,34,37]. These results indicate that local PT heating to a GNP-coated SEMS using NIR irradiation generates significant heat to block the granulation tissue formation. Photothermal local heating. BGNP-coated SEMS may also have the potential to prevent tissue ingrowth and overgrowth [38,39].

Tissue ingrowth resulting from stent-induced mechanical injury to the colon mucosal wall can be classified into three phases that occur over time: inflammation, proliferation, and remodeling [27,40–42]. According to literature, the proliferation phase begins 4–14 days after stent placement and increased angiogenesis [27,40,42]. This study clarified the therapeutic effect of heat by suppressing hypervascularization in the proliferation phase. Our suppression methodology had good results, and the therapeutic effect of heat suppressed the stent-induced granulation tissue formation after stent placement. To prevent long-term complications, our study showed that adequate local heating of BGNP-coated SEMS could prevent stent-induced tissue hyperplasia in the mouse colon.

There were some limitations to our study. First, the results may not accurately reflect the pathological mechanisms of granulation tissue formation in humans. Further studies are required to confirm the results and explore the effects of various aspects of local PT heating in animal experiments. Second, although representative markers of angiogenesis were evaluated, more precise pathological markers should be applied for further insight into the effect of local PT heating. Third, although the differences in the variables were of statistical significance, the sample size was small. However, the differences in the variables between the two groups are indisputable Finally, the experiment ended after a follow-up of only 4 weeks. Studies with long-term follow-ups are needed to identify changes caused by local heating. Nevertheless, our study supports a substantial therapeutic effect based on the results of endoscopic and pathological evaluations.

Despite their low complications and high technical success rates, many stent technologies have limited long-term stent patency. Local PT heating may be a valuable option for suppressing the stent-induced granulation tissue formation and tumor ingrowth and/or overgrowth in patients with benign and malignant obstructions. This stented mouse colon model enables the evaluation of the granulation formation mechanism. In conclusion, local PT heating suppresses granulation tissue formation after stent placement in the mouse colon.

## Supporting information

S1 Table Granulation tissue area, the thickness of submucosal fibrosis, and VEGF positivity in both groups after stent placement. (XLSX)
